# Placental Morphology and Metabolomic Profile in Uncomplicated Metabolically Healthy Obese Pregnancy

**DOI:** 10.3390/biomedicines13092149

**Published:** 2025-09-03

**Authors:** Ousseynou Sarr, Akasham Rajagopaul, Shuang Zhao, Xiaohang Wang, David Grynspan, Genevieve Eastabrook, Liang Li, Timothy R. H. Regnault, Barbra de Vrijer

**Affiliations:** 1 Department of Physiology and Pharmacology, Schulich School of Medicine and Dentistry, London, ON N6A 5C1, Canada; osarr@uwo.ca (O.S.); arajago2@uwo.ca (A.R.); tim.regnault@uwo.ca (T.R.H.R.); 2 The Metabolomics Innovation Centre (TMIC), University of Alberta, Edmonton, AB T6G 2G2, Canada; szhao1@ualberta.ca (S.Z.); xi14@ualberta.ca (X.W.); liang.li@ualberta.ca (L.L.); 3 Department of Pathology and Laboratory Medicine, University of British Columbia, Vancouver, BC V6T 2B5, Canada; david.grynspan@interiorhealth.ca; 4 Department of Obstetrics and Gynaecology, Schulich School of Medicine and Dentistry, London, ON N6A 5C1, Canada; genevieve.eastabrook@lhsc.on.ca; 5 Children’s Health Research Institute, London, ON N6C2R5, Canada; 6 Department of Chemistry, University of Alberta, Edmonton, AB T6G 2G2, Canada

**Keywords:** placenta, pregnancy, MHO, body mass index, metabolomics, LC-MS

## Abstract

**Background/Objectives:** Individuals with metabolically healthy obesity (MHO) and metabolically unhealthy obesity (MUO) in pregnancy are two distinct cardiometabolic populations, each potentially necessitating alternative clinical management. However, our understanding of the unique physiological effects of uncomplicated MHO on fetoplacental growth and metabolism remains limited. In this study, we aimed to identify changes in placental morphology and metabolites associated with maternal obesity, independent of pregnancy-related cardiometabolic complications. **Methods:** Placentae from women with a prepregnancy body mass index (BMI) < 25 kg/m^2^ (control; *n* = 15) and women with MHO (prepregnancy BMI > 30 kg/m^2^ with no cardiometabolic diseases; *n* = 15) were analyzed for indices of placental growth and untargeted metabolomics. Complementary assessments were conducted on proinflammatory genes and antioxidant defense system genes, proteins, and enzymes, along with lipid peroxidation markers. **Results:** Clear placentomegaly without histopathological changes was observed in uncomplicated MHO pregnancies. The metabolite 3-aminoisobutanoic acid emerged as the top-ranked feature distinguishing placentae from MHO individuals from control placentae, and changes in the cysteine, methionine, and vitamin B6 metabolism pathways were among the most distinct differences identified. **Conclusions:** These findings illustrate an altered placental morphology and metabolomic profile specific to uncomplicated MHO, offering new insights into how obesity, without cardiometabolic complications, may influence fetoplacental growth and metabolism. They may also represent a crucial first step towards marker identification for MHO pregnancy and underscore the importance of alternative care pathways when obesity is present but metabolic comorbidities are absent.

## 1. Introduction

Obesity, defined as abnormal or excessive body fat and measured by a body mass index (BMI) ≥ 30 kg/m^2^, is traditionally associated with adverse metabolic health outcomes and has become a global epidemic, posing a significant challenge to public health [1,2]. It is linked to a variety of metabolic and cardiovascular complications, including insulin resistance, type 2 diabetes, high blood pressure, and abnormal blood lipid levels [1]. However, evidence indicates that obesity does not always lead to adverse metabolic outcomes since BMI per se does not take into account the heterogeneity of body fat distribution, which is a key driver of cardiometabolic risk associated with obesity [3,4]. Around 10–30% of people with obesity are a metabolically healthy, that is, they lack overt cardiometabolic abnormalities [2]. This condition is referred to as ‘metabolically healthy obesity’ (MHO), which contrasts a metabolically unhealthy state, characterized by the presence of metabolic complications such as hypertension or diabetes (metabolically unhealthy obesity; (MUO)) [2]. Although there is no standardized definition of MHO, it generally refers to the absence of metabolic and cardiovascular diseases, including type 2 diabetes, dyslipidemia, hypertension, insulin resistance, and atherosclerotic cardiovascular disease in a person with obesity (BMI ≥ 30 kg/m^2^) [2,5].

Obesity complicating pregnancy is of particular concern, as an escalating number of women of reproductive age are now being categorized as obese [6]. It is estimated that obesity affects up to 25% of pregnant women in the Western world [7]. Maternal obesity is linked to a higher likelihood of obstetrical complications, including gestational diabetes mellitus (GDM), gestational hypertension, preeclampsia, preterm delivery, a large for gestational age (LGA) baby, caesarean section and, an increased risk of neonatal morbidity and mortality [8]. In addition to potentially experiencing negative immediate consequences, both the obese mother and her child are at an increased risk of developing cardiovascular, metabolic, and neurological disorders post-pregnancy later in life [9,10,11,12]. During pregnancy, maternal obesity with a metabolic comorbidity is referred to as MUO, similar to the non-pregnant population [9]. However, it is important to note that not all pregnant women with obesity experience metabolic dysfunction. In fact, a significant number are classified as having MHO [13] and do not exhibit the cardiometabolic complications typically associated with maternal obesity [13,14,15], even though they deliver LGA babies and have increased placental size [16], similar to women with MUO pregnancies [17].

The placenta plays a crucial role in the development and growth of the fetus, and changes in placental development and growth are associated with poor maternal, newborn, and offspring outcomes [18,19]. Indeed, changes in measures of placental growth, including the birth weight/placental weight (BPW) and birth weight/placental volume (BPV) ratios, which are measures of placental insufficiency, are observed in conjunction with adverse outcomes such as preeclampsia and GDM and can be used as predictors of pregnancy complications [20,21,22]. Furthermore, in a pregnancy with both obesity and GDM, the placenta is relatively large for the size of the fetus, This results in a relatively small BPW ratio, which is suggestive of reduced placental efficiency in these pregnancies [23]. This pattern of reduced placental efficiency is distinct from associations observed when considering fetal birth weight or placental weight in isolation [22]. Moreover, measures of the placenta itself have been associated with altered metabolism and as indicators/readouts of the in utero environment. Recent findings have revealed several links between placental morphometric features, such as weight, length, width, and thickness, with diseases experienced early and later in life [24,25], including under conditions such as gestational diabetes and preeclampsia, with the latter supported by histomorphometric studies focused on placental vasculature rather than overall placental size [26,27]. In particular, placental breadth is associated with neonatal body size and may be an important indicator of placental nutrient transport capacity [28]. In addition, the surface area and thickness of the placenta are proportional to the number of uterine spiral arteries that provide placental oxygenation and nutrition and the extent of villous branching, respectively [29,30,31].

Inflammation and the impact of oxidative stress are key regulators of these outcomes in pregnancy complicated by obesity [32]. Oxidative stress refers to an imbalance between the production of reactive oxygen/nitrogen species and the body’s ability to detoxify these reactive intermediates or repair the resulting damage [33]. In response to oxidative stress, the body also initiates inflammation, a defense mechanism that removes harmful or foreign stimuli and begins the healing process [34]. Changes in placental morphometric features may be influenced by placental inflammation, oxidative stress, and shifts in placental metabolism linked to obesity itself, specifically MHO, regardless of cardiometabolic comorbidities. Traditionally, placental oxidative stress, inflammation, and metabolic alterations have been assessed through histological analysis and targeted biochemical or cytokine assays. However, recent advances have introduced large-scale, untargeted metabolomic screening to provide a more comprehensive understanding of the global impact of obesity, especially MHO, on placental function.

The metabolome, the complete set of small-molecule chemicals (metabolites) found in a cell, cellular organelle, organ, tissue, or biofluid, is best studied using a comprehensive approach called metabolomics [35]. Metabolomics is used to identify phenotypical groups with specific metabolic profiles, and its application to various pregnancy conditions is on the rise [36]. Studies of placental metabolomics during uncomplicated and complicated obese pregnancies with optimal prenatal care (specific nutritional advice and recommendations for weight gain in pregnancy) have identified specific metabolomic signatures in placentae from people with obesity [37,38]. These studies mostly investigated clinical obesity in general, without differentiating between metabolically healthy and unhealthy phenotypes. A better understanding of how obesity without comorbidities, i.e., uncomplicated MHO, may alter placental morphology and the assessment of the metabolomic profile could help define baseline changes in placental growth and function [13,15,16]. In this study, we recruited women with MHO to better isolate the direct effects of obesity on placental development, independent of confounding metabolic comorbidities. By enabling a clear distinction between obesity-specific effects and those arising from MUO, in which cardiometabolic disorders independently influence placental function, this approach may inform more accurate risk stratification and personalized care in clinical settings.

This study accordingly examines changes in placental morphology and metabolomic profile related to uncomplicated MHO pregnancy. The primary aim was to analyze placental growth and metabolites at term in uncomplicated MHO pregnancies using liquid chromatography–mass spectrometry (LC–MS) and to evaluate placental changes in proinflammatory genes and antioxidant defense system genes, proteins, and enzymes, along with lipid peroxidation markers, in comparison to healthy controls. It was hypothesized that these findings would clarify how obesity without cardiometabolic comorbidities may modulate fetoplacental growth and metabolism. Furthermore, these findings could provide an essential initial step in identifying metabolomic markers for MHO and in guiding alternative clinical care pathways for pregnancies complicated by obesity in the absence of overt metabolic dysfunction.

## 2. Materials and Methods

### 2.1. Population

This study’s protocol was approved by Western University’s Human Subjects Research Ethics Board (REB# 106663). Pregnant women (normal weight; 18 kg/m^2^ ≤ prepregnancy BMI < 25 kg/m^2^, (control (C); *n* = 15) and pregnant women with obesity; (prepregnancy BMI > 30 kg/m^2^ (MHO), *n* = 15) were recruited at the time of their planned caesarean delivery without labor at term (>37 weeks’ gestational age, GA). Exclusion criteria were multiple pregnancy; maternal age < 18 or ≥40 years at estimated due date, maternal hypertensive disorders of pregnancy, gestational diabetes, or other significant medical conditions predisposing to placental dysfunction (renal, autoimmune disease); and prepregnancy BMI < 18 kg/m^2^ or prepregnancy BMI between 25 and 30 kg/m^2^. Hypertension in pregnancy was defined as at least two instances of a systolic blood pressure (BP) of ≥140 mmHg and/or a diastolic BP of ≥90 mmHg after 20 weeks of pregnancy in patients with an initially normal blood pressure. Preeclampsia was defined as de novo hypertension after 20 weeks of gestation in combination with proteinuria (serum urate level < 340 μmol/L). Gestational diabetes was excluded with a glucose challenge or tolerance test (GCT/GTT) (>11.1 mmol/L and 5.3/10.6/8.3 mmol/L, respectively) during the second trimester. Maternal clinical characteristics and obstetrical and neonatal outcome data were collected and previously described by Cohen et al. [16]. According to the exclusion criteria and maternal clinical characteristics, the group with obesity was classified as having metabolically healthy obesity (MHO). This group included participants with a BMI > 30 kg/m^2^ and no evidence of prepregnancy or pregnancy-onset cardiometabolic disease, such as gestational diabetes mellitus (GDM), hypertension, or preeclampsia, as determined by clinical screening, medical history, and pregnancy history. Additionally, the MHO group had normal liver function test results (i.e., no metabolic liver disease) and showed no signs of altered inflammation or impaired renal function, with normal uric acid levels.

### 2.2. Placental Tissue Collection

Placentae were collected at elective caesarean sections and the placental weight, length, thickness, breadth, and surface area were recorded. Placental tissue samples (*n* = 15 in each group) were collected from three different areas (center, periphery (1–2 cm from the placental edge), and middle (between these two sections)) within 30 min of delivery, snap-frozen in liquid nitrogen, and stored at −80 °C as previously reported [16].

### 2.3. Placental Histology

Formalin-fixed paraffin-embedded placental tissues were sectioned and stained with hematoxylin and eosin (Molecular Pathology, Robarts Research Institute). Stained slides were scanned using Aperio ScanScope CS (Leica, Microsystems CMS GmbH, Wetzlar, Germany). Slides of the peripheral, middle, and central regions of each MHO and control placenta were examined by a clinical pathologist (DG), in a blinded manner, and histopathological features recorded and graded according to the following scoring system: distal villous hypoplasia (none, focal, or diffuse), increased syncytial knots (none or present), chorangiosis (none or present), delayed villous maturation (none, focal, or diffuse), avascular fibrotic villi (none or small, intermediate, or large foci), increased focal perivillous fibrin deposition (none or present), massive perivillous fibrin deposition (none or diffuse), maternal floor infarct pattern (none or present), intervillous thrombi (none or present), and chronic inflammation (none, low-grade, or high-grade). The distributions of these different placental histopathological features are represented as frequency and percentage as described previously [39].

### 2.4. Untargeted Metabolomics Using Chemical Isotope Labeling (CIL) GC-MS

Except those specifically stated, all chemicals and reagents were purchased from Sigma-Aldrich (Markham, ON, Canada). Liquid chromatography–mass spectrometry (LC-MS) grade water, acetonitrile, methanol, and formic acid were purchased from Canadian Life Sciences (Peterborough, ON, Canada). For in-depth placental metabolomics, high-performance LC–MS was performed at The Metabolomic Innovation Centre (TMIC), Department of Chemistry, University of Alberta (Edmonton, AB, Canada) according to the published analytical workflow [40]. The workflow for sample processing and metabolomics analysis is presented in Supplementary Figure S1. Placental samples from the different placental regions were individually processed during the analysis.

The placental samples were homogenized in methanol, dichloromethane (DCM), and water and metabolites were extracted as previously described [41]. The samples were randomized prior to any procedure to eliminate potential batch variations in the analysis. Sample normalization was carried out by measuring the total metabolite concentration in each sample [42,43]. A proprietary metabolome quantification kit from Nova Medical Testing Inc. (Product Number: NMT-6001-KT) was used to measure the total concentration in a 25 µL aliquot of sample extract. Water was added to adjust the concentration of all samples to 1.2 mM.

Chemical isotope labeling was performed for each sample according to the standard operating procedures (SOPs) provided with the labeling kits (Nova Medical Testing Inc., Edmonton, AB, Canada). Each kit contains isotopic labeling reagents and additional components required for the derivatization reaction. Briefly, the ^12^C_2_-labeling reagent was added to individual samples, while the ^13^C_2_-labeling reagent was added to a pooled sample used as the reference. The additional reagents were then added in the sequence specified in the SOPs. The mixtures were thoroughly vortexed and incubated in an oven to complete the chemical derivatization reaction. For the labeling experiment, a 25 µL aliquot of sample was used. With these kits, the amine/phenol metabolites were labeled using dansylation [44].

After labeling, each individual ^12^C_2_-labeled sample was mixed with an equal volume of a ^13^C_2_-labeled reference sample. The mixture was then injected into an LC-MS system for analysis. Prior to LC-MS analysis of the entire sample set, a quality control (QC) sample was prepared with an equal volume mix of ^12^C_2_-labeled and ^13^C_2_-labeled pooled samples. QC samples were run at an interval of one QC injection after ten sample injections. All LC-MS analyses were carried out on an Agilent 1290 LC linked to a Bruker Impact II QTOF Mass Spectrometer. The column used was an Agilent eclipse plus a reversed-phase C18 column (150 × 2.1 mm, 1.8 µm particle size), and the column oven temperature was 40 °C. Mobile phase A was 0.1% (*v*/*v*) formic acid in water, and mobile phase B was 0.1% (*v*/*v*) formic acid in acetonitrile. The gradient setting was t = 0 min, 25% B; t = 10 min, 99% B; t = 15 min, 99% B; t = 15.1 min, 25% B; and t = 18 min, 25% B. The flow rate was 400 µL/min. The mass spectral acquisition rate was 1 Hz, with an m/z range from 220 to 1000.

All raw LC-MS data were first converted to .csv files using DataAnalysis 4.4 (Bruker Daltonics, Bremen, Germany). The exported files were uploaded to IsoMS Pro 1.2.12 (Nova Medical Testing Inc.) for data processing and metabolite identification. ^12^C-/^13^C-peak pairs in each sample were first extracted, and the peak intensity ratio was calculated for each peak pair [45]. In this step, all redundant information was filtered out (e.g., adduct ions, dimers)to retain one peak pair for each metabolite. The same peak pairs (i.e., metabolite) from different samples were then aligned, and missing ratio values were filled in by the software. Data cleansing was carried out to remove peak pairs that originated from blank samples and those that were not present in at least 80.0% of samples in any group. Data were then normalized using the ratio of total useful signal, which was calculated as the sum of all useful ^12^C-peaks over the sum of all useful ^13^C-peaks and enabled post-acquisition normalization [43].

Metabolite identification was carried out using a three-tiered approach against NovaMT Metabolite Databases 2.0 (Nova Medical Testing Inc.) [40,41]. In tier 1, peak pairs were searched against a labeled metabolite library (CIL Library), which was constructed using isotope-labeled authentic standards. Metabolites were considered positively identified based on accurate mass and retention time matching. In tier 2, the remaining peak pairs were searched against a linked identity library (LI Library), which includes over 9000 pathway-related metabolites, providing high-confidence putative identification results based on accurate mass and predicted retention time matches. In tier 3, any remaining peak pairs were searched, based on accurate mass matching, against the MyCompoundID (MCID) library (www.mycompoundid.org, accessed on 6 July 2021), which is composed of 8021 known human endogenous metabolites (zero-reaction library) and their predicted metabolic products from one metabolic reaction (375,809 compounds) (one-reaction library) and two metabolic reactions (10,583,901 compounds) (two-reaction library) [46].

### 2.5. RNA Extraction and Real-Time PCR

Tissue samples from the center, periphery, and middle of the placenta from each subject were pooled and ground prior to real-time PCR and spectrophotometric analyses. Total RNA was extracted from 50 mg of placental tissue using 1 mL of Trizol (Invitrogen, Burlington, ON, Canada) and the E.Z.N.A Total RNA kit I, following the manufacturer’s instructions (OMEGA bio-tek, Norcross, GA, USA). The quantity of extracted RNA was assessed using a NanoDrop spectrophotometer (Thermo Fisher Scientific, Waltham, MA, USA), and RNA quality and integrity were evaluated via the A260/A280 ratio and agarose gel electrophoresis. One microgram of RNA was reverse transcribed into cDNA using the M-MLV Reverse Transcriptase Kit (Thermo Fisher Scientific) following the manufacturer’s instructions. Real-time PCR was performed using the SensiFAST SYBR No-ROX Kit (FroggaBio, Concord, ON, Canada). All primers (Supplementary Table S1) were purchased from Sigma-Aldrich (Oakville, ON, Canada). The specificity of the PCR products was assessed using melting curve analysis. Non-transcribed RNA and PCR reactions using water instead of a template showed no amplification. Gene expression analysis was conducted using the CFX Maestro software (Version 2.3, Bio-Rad, Mississauga, ON, Canada) with the 2^−ΔΔCt^ method [47]. Beta-actin and proteasome 20S subunit beta type 6 (PSMB6) were used as reference genes. The average Ct values of the target gene were normalized to the average beta-actin and PSMB6 Ct values of the same cDNA sample. Beta-actin and PSMB6 were determined to be suitable reference genes based on the GeNorm algorithm in the CFX Maestro software, with an average expression stability (Avg M value) of 0.68 for both genes.

### 2.6. Antioxidant Marker Assays

Placental tissues (50 mg) were placed in an ice-cold homogenization buffer (20 mM HEPES, 70 mM sucrose, 1 mM EGTA, and 210 mM mannitol). Each placental sample was homogenized in 500 μL of isolation buffer using a PRO Scientific Bio-Gen PRO200 Homogenizer. The resulting homogenate was divided into 2 parts and centrifuged at different speeds to yield the following supernatants: supernatant 1, containing superoxide dismutase (SOD), which was centrifuged at 1500× *g* for five minutes at 4 °C; supernatant 2 containing catalase (CAT), which was centrifuged at 10,000× *g* for 15 min at 4 °C. The enzyme activity of SOD (kit no. 706002, Cayman Chemical, Ann Arbor, MI, USA) and CAT (kit no. 707002, Cayman Chemical) in the placental homogenates was measured by spectrophotometric analysis following the kit instructions. Reduced glutathione (GSH), oxidized GSH (GSSG), and the GSH/GSSG ratio were determined in 50 mg of placental tissue homogenized in 1 mL of 2-(N-morpholino)ethanesulfonic acid (MES) buffer provided in the GSH assay kit (kit no. 703002, Cayman Chemical) and, following the kit instructions.

### 2.7. Measurement of Lipid Peroxidation Markers

Total 8-isoprostane levels in the placental tissues were measured using the 8-isoprostane enzyme-linked immunosorbent assay kit (kit no 516351, Cayman Chemical), following the manufacturer’s instructions. Briefly, 50 mg placenta samples were homogenized in 500 μL of 0.1 M phosphate buffer, pH 7.4, containing 1.4 mM EDTA and 0.005% butylated hydroxytoluene (BHT), using a PRO Scientific Bio-Gen PRO200 Homogenizer. After homogenization, the samples were centrifuged at 8000× *g* for 10 min to pellet particulate matter. After the removal of 20 μL for protein analysis, 300 μL of supernatant for each sample was subjected to 15% (*w*/*v*) KOH hydrolysis (with incubation for 1 h at 37 °C) for the measurement of total 8-isoprostanes. The samples were then neutralized by adding 600 μL of 1N HCl to each supernatant. Extraction was performed using SEP cartridge C18 columns (Thermo Fisher Scientific). Isoprostanes were eluted with methanol, dried under nitrogen, and reconstituted in 300 μL of ELISA buffer (1x). An aliquot of 50 μL of reconstituted isoprostane extract was assayed in duplicate. The result was expressed in pg/mg protein.

Placental malondialdehyde (MDA) levels were measured using the thiobarbituric acid reaction (TBARS Assay, no. 10009055, Cayman Chemical Company). Briefly, 25 mg of placenta was homogenized in 250 μL of RIPA buffer containing 10 mM Tris-Cl, pH 8; 140 mM NaCl; 1% triton X-100; 0.1% sodium deoxycholate; 0.1% sodium dodecyl sulfate; 1 mM EDTA; 1 mM Na_3_V0_4_; 25 mM NaF; 1 mM PMSF; 1 μg/mL leupeptin, and aprotinin. Supernatants were obtained after centrifugation at 1600× *g* for 10 min at 4 °C. The absorbance of the supernatant fraction was read at 530 nm, and results were expressed in nmol per mg of placental tissue protein.

### 2.8. Statistical Analysis

Comparisons of clinical data, placental mRNA expression, antioxidant defense system enzymes and proteins, lipid peroxidation markers, and Pearson correlations were performed using two-tailed unpaired *t*-tests. The association of placental histopathological features with MHO was analyzed using Fisher’s exact test (GraphPad Prism 9.0.0, GraphPad Software, LLC, Boston, MA, USA). Differences were considered statistically significant when *p* < 0.05. Cohen’s *d* index was calculated to assess the effect size in, both in the control and MHO groups. Cohen’s *d* evaluates the magnitude of a difference between two groups. Values of Cohen’s *d* index below 0.19, between 0.20 and 0.49, between 0.50 and 0.79, between 0.80 and 1.29, and above 1.30 are classified as insignificant, small, average, large, and very large effects, respectively [48]. For the analysis of categorical variables of placental histopathology, the effect size was evaluated using Cramer’s V index. Values between 0.0 and 0.1, between 0.1 and 0.20, between 0.20 and 0.40, between 0.40 and 0.60, between 0.60 and 0.80, and between 0.80 and 1.0 indicate negligeable, weak, moderate, relatively strong, strong, and very strong effects, respectively [49].

Metabolomics data were analyzed using partial least squares discriminant analysis (PLS-DA), univariate analysis (*t*-test, volcano plot), and pathway analysis on the platform MetaboAnalyst 5.0 (https://www.metaboanalyst.ca/MetaboAnalyst/upload/StatUploadView.xhtml; accessed on 7 December 2022). To reduce complexity and enhance interpretability, metabolomic data from the three placental zones were pooled prior to analysis. Although previous studies demonstrated regional metabolic heterogeneity within placental villous compartments [50], our preliminary analysis comparing the three placental anatomical zones within the control group cohort showed no significant differences. This pooling approach enabled a more holistic assessment of placental metabolic alterations between experimental groups while acknowledging that subtle regional variations may be masked. Raw data were normalized by autoscaling and then used for both univariate and multivariate analyses. The PLS-DA model was cross-validated by evaluating validation metrics including the predicted (Q^2^) and explained (R^2^) variances. The statistical significance of altered metabolites within the experimental groups was set to a false discovery rate (FDR)-corrected *p*-value < 0.25, raw *p*-value < 0.05, and fold change > 1.2 or <0.83. Variable importance in projection (VIP) ranking analysis was then performed to investigate a variable’s importance in the PLS-DA model. The VIP was used as a quantitative estimation of the discriminatory power of each individual metabolite. Metabolites with a VIP score of >1 were considered important in the PLS-DA model. Receiver operating characteristic curve (ROC) analysis was performed with SPSS 28.0 (IBM SPSS Statistics, Armonk, NY, USA) to calculate the area under the curve (AUC) to evaluate the accuracy of the altered metabolites in diagnosing MHO from placentae. An AUC of 0.5 indicates a poor diagnostic test, while a value of 1.0 indicates an ideal test. MetaboAnalyst pathway analysis was then performed on data for all the identified metabolites in tiers 1 and 2, via MetaboAnalyst 5.0, using the Human Metabolome Database (HMDB) and KEGG identifiers of metabolites, to identify the affected metabolic pathways (FDR < 0.05 and pathway impact > 0.1).

A complete list of all reagents, instruments, and kits is provided in Supplementary Table S2.

## 3. Results

### 3.1. Pregnancy and Placental Characteristics

The maternal and delivery characteristics of the cohort were previously reported by Cohen et al. [16]. Mean prepregnancy BMI was higher in the MHO group than the control (C) group (C: 21.1 ± 2.0 kg/m^2^ vs. MHO: 42.3 ± 7.6 kg/m^2^, *p* < 0.0001, Cohen’s *d* = 3.8). Birth weight was significantly higher in the MHO group than the C group (C: 3170.0 ± 403.4 g vs. MHO: 3795.3 ± 717.8 g, *p* < 0.01, Cohen’s *d* = 1.1). The placental characteristics of each group are provided in Table 1. The placentae were heavier in the MHO group (C: 592.3 ± 111.0 g vs. MHO: 762.0 ± 164.3 g, *p* < 0.01, Cohen’s *d* = 1.2), which was reflected in an increase in placental breadth (C: 15.1 ± 1.5 cm vs. MHO: 16.9 ± 2.0 cm), thickness (C: 1.5 ± 0.4 cm vs. MHO: 2.0 ± 0.4 cm), and surface area (C: 199.6 ± 34.9 cm^2^ vs. MHO: 243.7 ± 52.7 cm^2^) (for all measures, *p* < 0.05, Cohen’s *d* ≥ 1). The increase in placental length in the MHO group trended to significance (C: 16.7 ± 2.1 cm vs. MHO: 18.1 ± 2.0 cm, *p* = 0.068, Cohen’s *d* = 0.7). The fetal–placental weight ratio, a measure of placental efficiency [20,21], was not statistically different between the two groups (C: 5.0 ± 0.1 vs. MHO: 5.4 ± 0.2, *p* = 0.2821, Cohen’s *d* = 2.5). Despite the morphometric changes in the placenta, the placental histopathology parameters were not significantly different between the MHO and control groups (*p* > 0.999, Cramer’s < 0.3, Table 1). However, minor tendencies toward increased delayed villous maturation and chorangiosis, although not statistically significant, were noted.

### 3.2. Changes in the Metabolome in MHO

**Figure 1:**
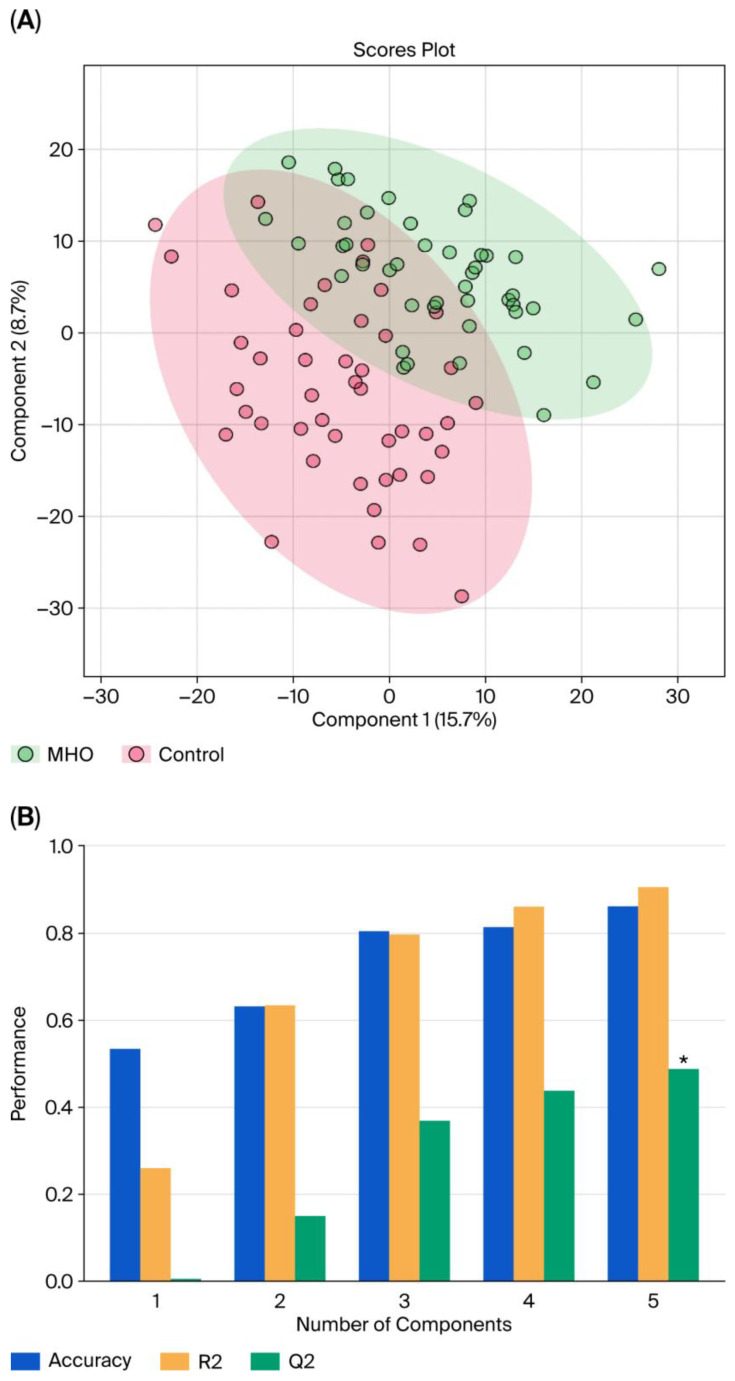
**Partial least squares discriminant analysis (PLS-DA) of control and MHO groups.** (**A**) The PLS-DA 2D scores show a difference between the MHO and control groups. The red dots represent the MHO samples, while the green dots represent the controls. Data from the three areas of the placenta (center, periphery (1–2 cm from the placental edge), and middle (between these two sections)) and each set of three samples per placenta were pooled. (**B**) Cross-validation chart: PLS–DA classification performance showed that the best classifier was obtained using 5 components, considering the accuracy, variations (R2), and prediction of the model (Q2). The asterisk (*) denotes the statistically optimal number of components selected based on cross-validation (*p*-value < 0.05).

With LC–MS, 2808 metabolite peak pairs were detected in each sample. Data from each individual placental region (center, periphery, and middle) were analyzed separately within the control group. No significant differences were found among the three anatomical zones, allowing the three placental samples to be pooled for further analysis. Figure 1 displays the pooled PLS-DA results. Separation is observed between the two groups, with a degree of overlap between the women with MHO and control women (Figure 1A). This separation is estimated by the two quality parameters, R^2^ = 0.904 for the explained variation and Q^2^ = 0.487 for the predictive capability of the model (Figure 1B).

**Figure 2:**
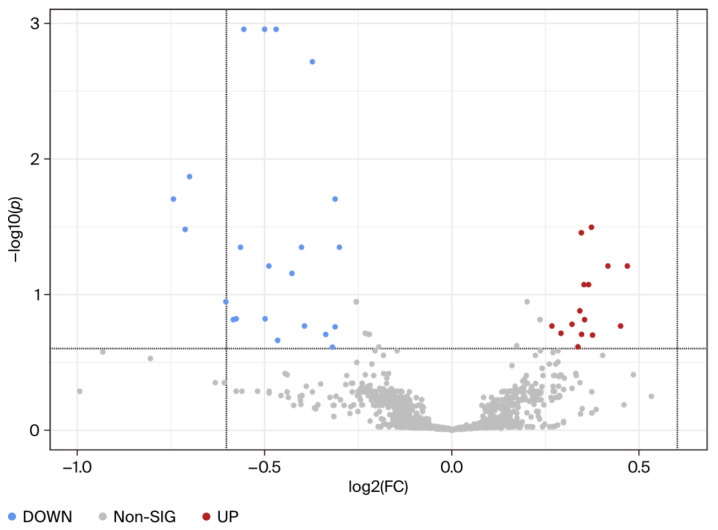
**Visualization of changes in metabolite patterns by volcano plots using univariate analysis.** Volcano plots show the statistically significantly altered metabolites (FDR-corrected *p*-value < 0.25, raw *p*-value < 0.05, and FC > 1.2 or <0.83). *n* = 15 per group with data from combined central, peripheral, and middle placental **samples from each individual**. The x-axis indicates log2 (fold change) vs. the MHO group, and the y-axis indicates −log10 (*p*-value). The levels of 22 metabolites were downregulated (blue) and 15 were upregulated (red) in the MHO group compared to the control group.

Using univariate analysis, the abundance of each metabolite peak pair (intensity) was compared between the MHO and control groups. Of the 2808 recorded metabolite peak pairs, 561 were successfully identified in tier 1 (192) and tier 2 (369) (Supplementary Table S3) and 37 were found to be differentially abundant in the MHO and control groups (fold change > 1.2 or <0.83, and FDR-*p*-value < 0.25 and raw *p*-value < 0.05) (Figure 2).

Among the 37 metabolite peak pairs, abundance was low for 22 and high for 15 in the MHO group. Of these differentially abundant peak pairs, 10 were successfully identified in tiers 1 and 2 and are displayed in Table 2.

**Figure 3:**
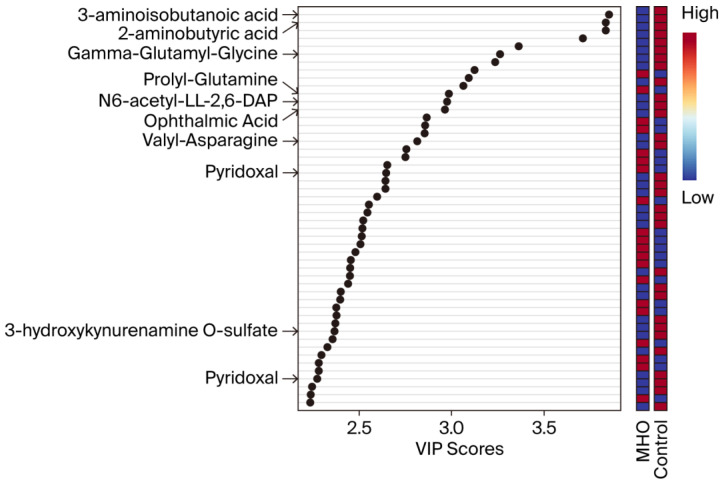
**Variable importance in projection (VIP) scores of the PLS-DA.** VIP scores indicate the top 50 metabolites contributing to the separation of metabolic profiles in the control vs. MHO groups. The relative abundance of metabolites is indicated by a colored scale ranging from blue to red representing low and high, respectively. Identified metabolites with a high confidence level are displayed on the left side of the chart.

VIP scores were used to identify the metabolites contributing the most to changes in the placentae from the MHO group (Figure 3). The top 50 metabolite peak pairs with the highest significance in group discrimination (VIP > 1) identified metabolites including 3-aminoisobutanoic acid, 2-aminobutyric acid, gamma-glutamyl-glycine, ophthalmic acid, prolyl-glutamine, N6-acetyl-LL-2,6-diaminoheptanedioic acid (N6-acetyl-LL-2,6-DAP), valyl-asparagine, pyridoxal, 3-hydroxykynurenamine O-sulfate, and pyridoxal phosphate that were downregulated in MHO. These had VIP scores > 2 and were considered relevant metabolites.

**Figure 4:**
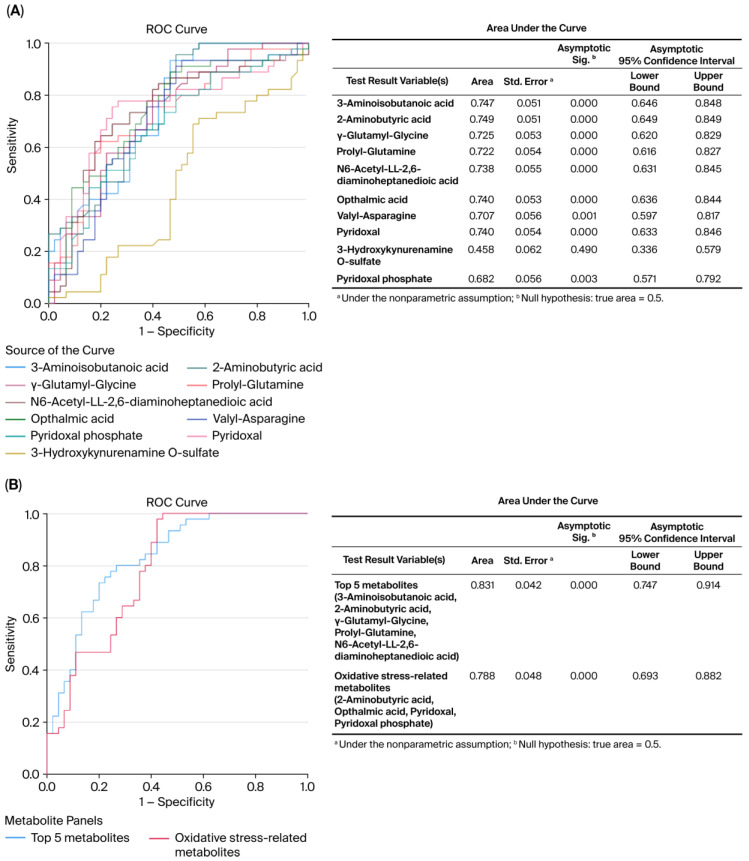
**Receiver operating characteristic (ROC) curves constructed with the logistic regression model and area under the curve (AUC) tables for discrimination between placentae from the control and MHO groups.** (**A**) ROC curves and AUC table showing the accuracy of individual metabolites to diagnose MHO from placentae. (**B**) ROC curves and AUC table showing the accuracy of combined metabolites to diagnose MHO from placentae.

ROC curve analysis revealed the diagnostic accuracy of these metabolites in discriminating between the control and MHO groups. Each identified metabolite, with the exception of 3-hydroxykynurenamine and pyridoxal phosphate, showed high diagnostic accuracy (AUC ≥ 0.7, *p* < 0.01) (Figure 4A). The diagnostic accuracy of individual top-ranked metabolites based on VIP scores including 3-aminoisobutanoic acid, 2-aminobutyric acid, gamma-glutamyl-glycine, and N6-acetyl-LL-2,6-diaminoheptanedioic was lower than the curve obtained from their combination (AUC = 0.831, *p* < 0.0001) (Figure 4B). Similar observations were made for metabolites related to oxidative stress, including 2-aminobutyric acid, ophthalmic acid, pyridoxal, and pyridoxal phosphate (AUC = 0.788, *p* < 0.0001) (Figure 4B).

**Figure 5:**
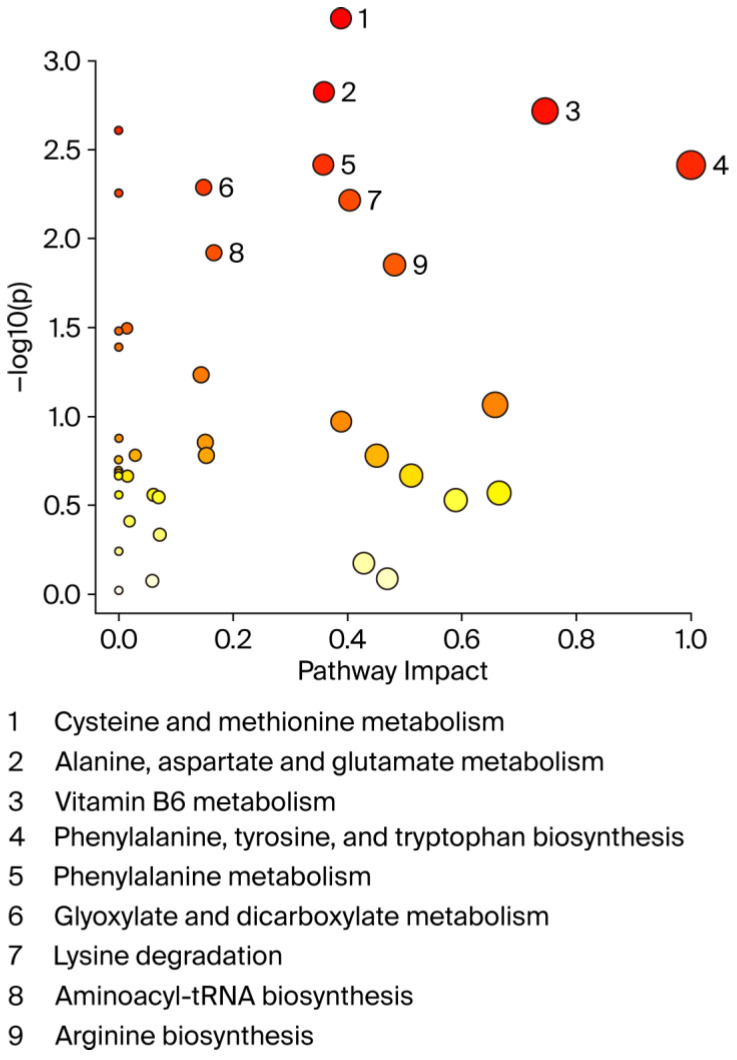
**Pathway analysis by MetaboAnalyst.** X-axis shows pathway impact values from pathway topology analysis; y-axis displays matched pathways from pathway enrichment analysis arranged by log (*p*-value). Red indicates most significant effects according to *p*-value. Node size is determined by pathway impact value. Nine altered pathways (FDR < 0.05 and pathway impact > 0.1) are displayed.

A graphical summary of the identified pathways and their relative impact is shown in Figure 5. Overall, the perturbed pathways were closely related to amino acid and vitamin B6 metabolism and included cysteine and methionine metabolism, alanine, aspartate, and glutamate metabolism, phenylalanine, tyrosine, and tryptophan biosynthesis, phenylalanine metabolism, glyoxylate and dicarboxylate metabolism, lysine degradation, aminoacyl-tRNA biosynthesis, and arginine biosynthesis (FDR < 0.05; impact values > 0.1).

**Figure 6:**
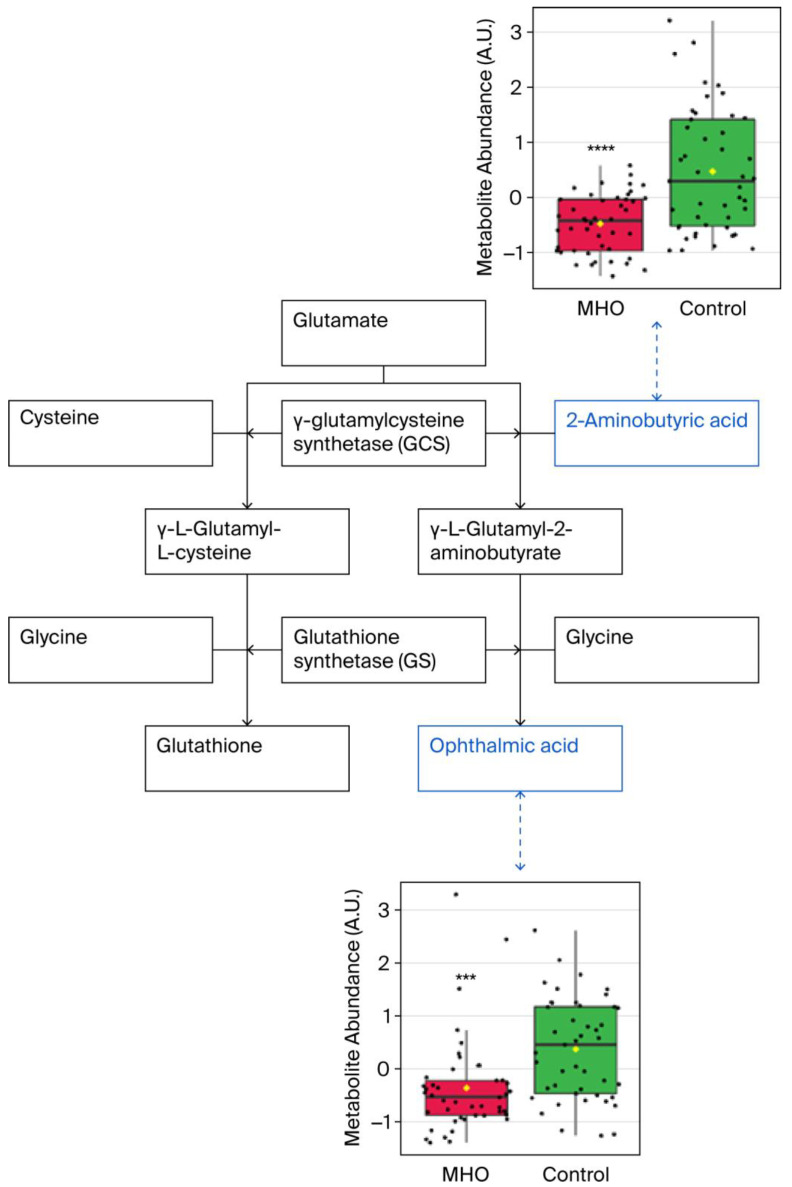
**Observed metabolite changes mapped onto maintained glutathione and reduced ophthalmic acid biosynthesis in placentae from the MHO group.** Box plots of 2-aminobutyric acid and ophthalmic acid abundance (metabolite peak pair intensity) in the MHO (red boxes) and control (green boxes) groups. **** and *** indicate significant differences between the MHO and control placentae; *p*-values < 0.0001 and 0.001, respectively. *n* = 15 per group.

Significant downregulation of the glutathione analog ophthalmic acid and its precursor 2-aminobutyric acid were observed in, the cysteine and methionine metabolism pathway, which was the most altered metabolic pathway (Figure 6).

**Figure 7:**
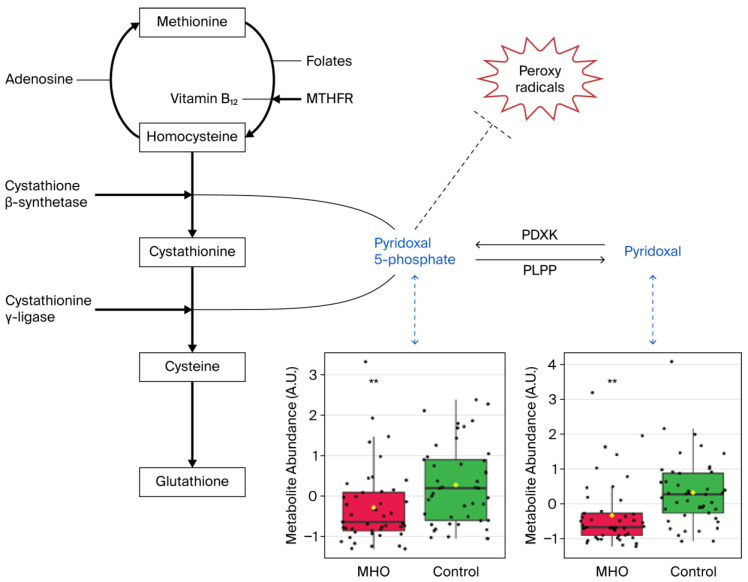
**Metabolite changes revealed decreases in vitamin B6 forms in the placentae from the MHO group.** Pyridoxal phosphate (pyridoxal 5′-phosphate) is the active form of vitamin B6 (pyridoxine or pyridoxal) and acts as a coenzyme in transsulfuration and glutathione biosynthesis. Pyridoxal phosphate may also directly react with peroxy radicals and thereby scavenge radicals. Box plots of pyridoxal and pyridoxal phosphate abundance (metabolite peak pair intensity) in the MHO (red boxes) and control (green boxes) groups are displayed. ** significant difference between placentae from women with MHO and controls, *p* < 0.01. *n* = 15 per group. MTHFR = methionine synthase reductase, PDXK = pyridoxal kinase, PLPP = pyridoxal 5-phosphate phosphatase.

Significant decreases in pyridoxal and pyridoxal phosphate, which are known to be involved in the defense against cellular oxidative stress, were responsible for vitamin B6 metabolism pathway alterations in the MHO group (Figure 7).

### 3.3. Relationship Between Metabolite Changes and Placental Measurements

To understand the relationship between metabolite changes and placental characteristics, we performed linear correlations between metabolite abundance and placental weight, thickness, breadth, and surface area in the two study groups (Table 3). Reductions in the abundance of 3-aminoisobutanoic acid and 2-aminobutyric acid were correlated with increased placental weight, thickness, and breadth (*p* < 0.01). while reductions in the abundance of gamma-glutamyl-glycine and ophthalmic acid were correlated only with increased placental thickness and breadth (*p* < 0.05). The low abundance of N6-acetyl-LL-2,6-DAP was, however, correlated with increased placental weight and thickness. Further, the abundance of valyl-asparagine and pyridoxal decreased as placental weight and breadth increased (*p* ≤ 0.05). Finally, placental thickness and weight increased while the abundance of prolyl-glutamine and pyridoxal phosphate decreased, respectively (*p* < 0.05).

### 3.4. Placental Antioxidant Defense and Proinflammatory Markers

**Figure 8:**
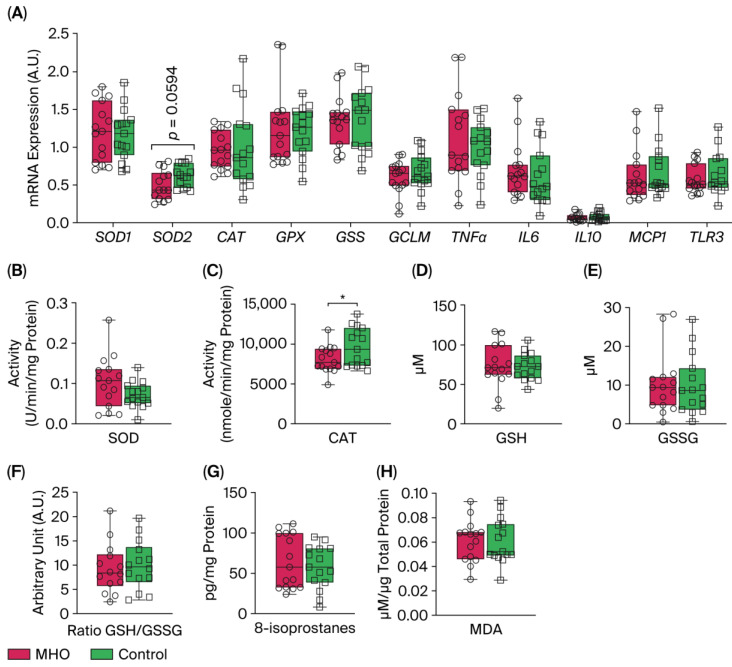
**Antioxidant and proinflammatory genes, antioxidant defense system enzymes and proteins, and lipid peroxidation markers**. Panel (**A**) shows mRNA expression of *superoxide dismutase 1* and *2* (*SOD1* and *2*), *catalase* (*CAT*), *glutathione* (*GPX*), *glutathione synthetase* (*GSS*), and *glutamate-cysteine ligase modifier subunit* (*GCLM*) and inflammatory markers *tumor necrosis factor α* (*TNFα*), *interleukin 6* (*IL6*), *interleukin 10* (*IL10*), *monocyte chemoattractant protein 1* (*MCP1*), and *Toll-like receptor 3* (*TLR3*). Panels (**B**,**C**) indicate placental SOD and CAT activity, respectively. Panels (**D**–**F**) show reduced glutathione (GSH), oxidized GSH (GSSG), and GSH/GSSG ratio, respectively. Panels (**G**,**H**) show placental total 8-isoprostanes and malondialdehyde (MDA) levels, respectively. Data are box plots with min to max of 14–15 individuals per group. * indicates *p*  <  0.05 when comparing MHO vs. control groups.

mRNA levels of expression of *superoxide dismutase 1* (*SOD1*), *catalase* (*CAT*)*, glutathione peroxidase* (*GPX*)*, Glutathione synthetase* (*GSS*), and *glutamate-cysteine ligase modifier subunit* (*GCLM*) were not affected in the MHO group compared to the control group (*p* > 0.10, Cohen’s *d* **≤** 0.3, Figure 8A). Conversely, a moderate decrease in the expression of *SOD2* was observed in the MHO group (−21%, *p* = 0.0594, Cohen’s *d =* 0.7). The mRNA expression of proinflammatory markers including *tumor necrosis factor α* (*TNFα*), *interleukin 6, 10* (*IL6, IL10*), *monocyte chemoattractant protein 1* (*MCP1*), and *Toll-like receptor 3* (*TLR3*) was not affected in the MHO group (*p* > 0.10, Cohen’s *d* < 0.3, Figure 8A). While SOD activity was not significantly impacted (*p* = 0.10, Cohen’s *d* = 0.6), CAT activity was lower in the MHO group (−16%, *p*  <  0.05, Cohen’s *d* **=** 0.7) (Figure 8B,C). GSH and GSSG concentrations and the ratio of GSH:GSSG did not differ between the MHO and control groups (*p* > 0.7, Cohen’s *d* **<** 0.2; Figure 8D–F).

### 3.5. Lipid Peroxidation Markers

The two universal parameters related to oxidative damage to lipids, 8-isoprostanes and malondialdehyde (MDA) levels, which are hallmarks of arachidonic acid and lipid peroxidation respectively, showed no significant changes in the MHO versus control groups (*p* > 0.5, Cohen’s *d* < 0.3; Figure 8G,H).

## 4. Discussion

This study’s primary objective was to determine whether pregnancies in women with MHO, who lack cardiometabolic comorbidities, exhibit distinct placental morphometry and metabolomic profiles compared to pregnancies in women of normal-weight. Our findings indicated that uncomplicated pregnancies with maternal obesity classified as MHO pregnancies were associated with large, thick, and heavy placentae. These morphometric changes were accompanied by altered metabolomic readouts, despite a lack of significant histopathological abnormalities. The metabolite 3-aminoisobutanoic acid was the top-ranked metabolite in differentiating placentae from MHO and control mothers, and the cysteine and methionine and vitamin B6 metabolism pathways were among the most distinct changes in the placentae from the MHO group. Additionally, biosynthesis of 2-aminobutyric acid, which serves as a marker of glutathione depletion [51], and the antioxidant forms of vitamin B6, pyridoxal, and pyridoxal phosphate [52,53], were reduced in the placentae from the MHO group. These changes occurred alongside a decline in the activity of the antioxidant defense system enzyme catalase; however, superoxide dismutase activity, glutathione levels, and lipid peroxidation markers remained consistent between control and MHO pregnancies. These pathway changes showed selective reductions in antioxidant defense system molecules, including vitamin B6 metabolites, though oxidative damage markers remained unchanged. These observations indicate that, alongside alterations in placental morphometric characteristics, there exists a specific metabolomic signature showing a selective and partial decline in the antioxidant response, without evidence of oxidative or tissue damage in the placentae from the MHO group, underscoring the nuanced nature of antioxidant regulation in this context.

The placenta plays a crucial role in intrauterine programming, and its phenotype has been shown to serve as a more effective indicator of disease risk later in life than other identifiers of exposure to suboptimal conditions during intrauterine development [54]. Recent findings revealed several links between placental morphometric features, such as weight, length, width, and thickness, and diseases early and later in life [24,25]. These gross morphometric characteristics are considered biologically linked to the functional capacity of the placenta and may impose mechanical or other constraints on the developing fetus [24]. Moreover, changes in placental morphometric characteristics are commonly associated with maternal obesity [23,55]. However, data on placental morphometric changes specifically associated with MHO pregnancy remain limited, despite MHO being the most common obesity phenotype during pregnancy [13]. The finding that MHO pregnancies show a significant increase in placental thickness, without the metabolic disturbances common to MUO, underscores the importance of distinguishing these subtypes. Our findings, however, contrast a report by Bianchi in which placental weight, diameter, and area were unchanged in obese pregnancies without GDM [23]. Notably, Bianchi et al. [23] also reported significantly less gestational weight gain in pregnancies with obesity, a pattern not observed in our cohort [16]. Our findings emphasize specific placental morphometric changes, suggesting that these features may be unique to obesity and not necessarily associated with conditions like GDM, thereby underscoring the importance of accounting for BMI in studies that assess pregnancy complications in which obesity is a significant risk factor.

In line with the increased placental breadth and surface area observed, the MHO pregnancies also resulted in infants with increased birth weight. Placental breadth is associated with neonatal body size, and breadth is an indicator of the transfer of nutrients from mother to fetus; the larger the breadth the greater the transfer [28]. Additionally, studies have shown that placental surface area is proportional to the number of uterine spiral arteries available for maternal blood supply, while placental thickness reflects the level of villous ramification and is strongly associated with fetal growth parameters [30,31]. Ratio data such as placental ratios, including birth weight/placental weight and birth weight/placental volume, have been used to improve our interpretation of single measures and suggested to predict pregnancy complications such as GDM and preeclampsia before delivery [22]. It is interesting to note that the birth weight/placental ratio was unchanged in the MHO pregnancies observed in this study, as previously reported in cases of obese pregnancy without GDM [23]. This difference possibly underscores the uncomplicated nature of the pregnancies studied, unlike obese + GDM pregnancies, in which the fetal/placental ratio is reduced [23]. The observed data, combined with observed placental morphometric changes, suggest compensatory and/or enhanced placental growth [56] to accommodate increased metabolic demands arising from accelerated fetal growth in these pregnancies, potentially acting as an early mechanism to support fetal nutrient transfer while also posing long-term metabolic or cardiovascular risks for the offspring.

While placental morphometric readouts afford a understanding of the in utero environment, placental metabolomics provides a comprehensive understanding of placental function and subsequent metabolism. In this study, a VIP plot of metabolomics analysis results highlighted that 3-aminoisobutanoic acid is the most crucial metabolite to differ between the placentae from the MHO and control groups. This metabolite, also known as 3-aminoisobutyrate or β-aminoisobutyric acid (BAIBA), appears to be involved in stimulating free fatty acid oxidation and glucose uptake in metabolically active tissues such as adipose tissue, liver, and skeletal muscle [57]. Moreover, 3-aminoisobutanoic acid is an AMPK/Akt-dependent modulator that normally restrains inflammation, mitigates oxidative stress, and supports mitochondrial metabolism [58]. Consistent with earlier reports, low levels of circulating 3-aminoisobutanoic acid observed in obesity [59] align with our findings, which showed reduced levels in placentae from the MHO group and an inverse correlation with placental weight, thickness, and breadth. Together with a partial decline in antioxidant response, its depletion suggests a subtle shift in placental redox and metabolic homeostasis in the placentae from the MHO group. Overall, these data highlight 3-aminoisobutanoic acid as a promising biomarker for placental adaptations in MHO pregnancy. Its inverse correlation with placental dimensions further supports its potential utility in predicting or diagnosing MHO at earlier gestational stages.

Reductions in ophthalmic acid and 2-aminobutyric acid also emerged as key features of the placentae from the MHO group, both correlating inversely with placental size. Ophthalmic acid, also known as ophthalmate, is synthesized in vivo from 2-aminobutyric acid through the same enzymatic machinery as glutathione, a key antioxidant tripeptide and one of the most abundant intracellular antioxidants [60]. Interestingly, ophthalmic acid lacks a reducing cysteine moiety and is therefore far more stable than glutathione [61,62]. Considered together, the altered cysteine and methionine metabolism pathway and the lack of change in both reduced and oxidized glutathione indicate a modified antioxidant profile suggestive of adaptation rather than overt deficiency in the placentae from the MHO group. This aligns with previous reports of a placental adaptation of the affected antioxidant response toward a nitric-oxide-induced alternative pathway and highlights changes in the reactive oxygen species/reactive nitrogen balance in order to mitigate potential adverse oxidative stress and reduce oxidative damage to preserve placental function in MHO pregnancy [15].

The vitamin B6 pathway is associated with protection against oxidative stress. It is a central pathway required for many processes, ranging from amino acid biosynthesis and catabolism, fatty acid biosynthesis and breakdown, and antioxidant mechanism, to the biosynthesis of neurotransmitters [52,53,63]. The metabolites pyridoxal and pyridoxal phosphate (PLP) are among the major forms of vitamin B6 [64], and, PLP is the active form of within cells [65]. In this study, significant disturbances in the vitamin B6 metabolism pathway, including reduced pyridoxal and PLP, were observed in the placentae from mothers with MHO. Bjørke-Monsen et al. demonstrated that prepregnancy BMI is inversely related to PLP [66] and postulated that lower pyridoxal and PLP levels may contribute to adverse pregnancy outcomes associated with maternal obesity, as an optimal micronutrient status is vital for normal fetal development [67]. In this sense, our data indicate that lower pyridoxal and PLP levels were linked to reduced placental weight and breadth in the placentae from the MHO group, suggesting a potential role in mediating adverse pregnancy outcomes.

Vitamin B6 also plays a significant role in preventing decreases in the antioxidant enzyme catalase in peripheral tissues in vivo and in vitro, by scavenging superoxide radicals [52,53]. Interestingly, paralleling lower levels of B6 isoforms (pyridoxal and PLP), a decrease in the activity of the antioxidant defense system enzyme catalase in placentae from the MHO group was observed. While these indicators suggest a potential selective and partial decline in the antioxidant response, protective mechanisms appear to have remained active, as there were no changes in malondialdehyde (MDA) and 8-isoprostane, downstream markers formed by the peroxidation of lipids and arachidonic acid, respectively [68]. This agrees with reports on full-term placentae from mothers with MHO showing decreased activity of catalase and SOD, key antioxidant defense system enzymes, and no significant change in MDA levels [15].

Crucially, our metabolomics data indicated alterations in vitamin B6 isoforms, specifically reduced levels of pyridoxal and PLP, which may diminish B6-dependent reactions such as transsulfuration and one-carbon metabolism, both of which are necessary for glutathione synthesis [69]. It is interesting to note, however, that in our study, glutathione synthesis was not diminished. This is consistent with other clinical evidence regarding marginal B6 deficiency that shows only a tendency for reduced glutathione synthesis in erythrocytes, with no change in glutathione concentrations in plasma or red blood cells [70], and with animal studies showing that moderate to severe B6 deficiency does not consistently limit hepatic glutathione generation [71]. In contrast, under conditions of severe vitamin B6 deficiency, antioxidant defenses are broadly compromised. For instance, B6-deficient rats exhibit decreased hepatic catalase and SOD activity along with a lower GSH/GSSG ratio, reflecting a weakened antioxidant capacity and increased lipid peroxidation [72]. Similarly, Cabrini et al. found that vitamin B6 deficiency lowered the reduced/oxidized glutathione ratio and increased TBARS production in liver and heart tissues [73]. Overall, our findings indicate a selective, partial reduction in the placental antioxidant response; without compromising glutathione synthesis despite impaired B6 metabolism. Building on these findings, the elevated levels of glutathione analogs (ophthalmic acid and 2-aminobutyric acid) and unchanged SOD activity further indicate a selective, partial reduction in the placental antioxidant response in the placentae from the MHO group.

In conjunction with this selective antioxidant landscape alteration, uric acid was detected without significant group differences, further supporting the existence of a finely tuned oxidative balance in the placentae from the MHO group due to its dual antioxidant and prooxidant roles [74]. These metabolic alterations most likely reflect mild impairments in redox homeostasis and antioxidant capacity, rather than a clear-cut deficiency. However, mild redox imbalances may disrupt placental function by interfering with redox-sensitive processes such as trophoblast invasion and vascular remodeling, which are essential for proper placental development and efficient nutrient transfer [75,76]. In support of these observations, previous proteome analyses in obese pregnancies reported dysregulation of antioxidant defense proteins, altered amino acid transporters, and imbalanced oxidative stress responses, particularly in pathways linked to B6 metabolism and redox regulation. For example, downregulation of catalase and SOD proteins [77], as well as upregulation of nutrient transporters such as the System A amino acid transporter, have been documented in maternal obesity [56], though stratification for MHO or MUO status has yet to be performed. Together, our data and previous studies suggest that moderate disruptions in vitamin B6 metabolism in the MHO setting may alter placental redox homeostasis, potentially affecting metabolic signaling and epigenetic regulation and thereby modifying trophic support to the fetus.

Given the close interplay between oxidative stress and inflammation in mediating placental dysfunction, it is also important to consider the role of inflammation in MHO pregnancies. Inflammation, in association with obesity, is known to be associated with placental dysfunction and pregnancy complications [78]. Elevated levels of inflammatory markers, such as interleukin 6 (IL6), IL8, IL1β, and monocyte chemotactic protein-1 (MCP-1), are observed in both maternal plasma and the placenta in cases of maternal obesity [79]. This cytokine profile is driven by several inflammatory pathways including Toll-like receptor 4 (TLR4) activation and the promotion of nuclear factor kappa light chain enhancer of activated B cells (NF-κB), leading to increased generation of reactive oxygen species and the secretion of proinflammatory cytokines such as IL6, IL8, MCP-1, tumor necrosis factor α (TNF-α), and IL1β [79]. It is of interest to note that in this study, no significant changes in the mRNA expression of proinflammatory markers *TNFα*, *IL6, IL10*, *MCP1*, and *TLR3* were observed in the placentae from the MHO group. This appears to contrast MUO pregnancies, in which inflammation is typically heightened [79] and mRNA expression of these proinflammatory markers is increased [56]. This suggests that metabolic health status may play a more critical role in shaping the placental inflammatory milieu than BMI alone.

The evaluation of receiver operating characteristic (ROC) curves is a standard method of comparing various predictors across a range of values. Beyond visual estimation, the calculation of the area under the curve and the application of test algorithms aid in identifying strong predictors [80]. Using the current dataset, 3-aminoisobutanoic acid, 2-aminobutyric acid, gamma-glutamyl-glycine, prolyl-glutamine, N6-acetyl-LL-2,6-DAP, ophthalmic acid, valyl-asparagine, and pyridoxal metabolites were effective in identifying placentae from mothers with MHO, with AUC values greater than or equal to 0.7. Notably, O-sulfate 3-aminoisobutanoic acid, 2-aminobutyric acid, prolyl-glutamine, and N6-acetyl-LL-2,6-diaminoheptanedioic or N6-acetyl-LL-2,6-DAP acid, along with valyl-asparagine, when combined, formed an outstanding group marker for identifying placentae from mothers with MHO, with AUC values of 0.8. Future development of predictive assays that incorporate these metabolites, either individually or in combination, could facilitate the identification of MHO pregnancies, thereby enabling more tailored clinical management approaches. While these findings offer promising biological insights, it is important to note that the identified metabolite panels are exploratory. Validation in larger, independent cohorts is necessary before these markers can be considered for clinical application.

The selection of study participants with no clinical diagnosis of preexisting disease or obesity-related pregnancy complications, all of whom underwent caesarean deliveries, ensures that the cohort represents pregnancies without cardiometabolic comorbidities. Standardizing the mode of delivery was intended to avoid labor-induced metabolic fluctuations and provide a clearer assessment of placental metabolites. However, it is important to note certain limitations. Specifically, the absence of comprehensive dietary data and of maternal blood profiling, including prooxidants and antioxidants that cross the placental barrier, limits our understanding, especially given that oxidative stress is a multifaceted process requiring cautious interpretation of a restricted set of biochemical markers. Future studies should incorporate maternal redox profiling and detailed nutritional assessments to better understand the maternal contributions to placental oxidative stress and metabolic programming. Although including only fasted caesarean deliveries controlled for labor-induced metabolic and inflammatory changes, future research should include placentae from both vaginal and elective caesarean section births. Maternal blood samples obtained both at term and postpartum would be helpful in assessing whether these findings hold in broader obstetric contexts. In addition, due to the relatively small sample size of the MHO and control groups, fetal sex-based analyses could not be performed, which may also limit the ability to detect more subtle metabolic differences. Another key limitation of this approach is that pooling the placental regions may have masked subtle zone-specific metabolic differences that could have biological relevance. Furthermore, this preliminary study did not evaluate, the impact of maternal MUO. The spectrum of obesity is generally considered a continuum with MHO eventually leading to MUO, and our MHO pregnancies may have been subclinical in their progress to MUO. Moreover, incorporating the analysis of supplementary matrices like plasma samples will be crucial to further enhancing the clinical applicability of these findings. Finally, integrating the assessment of plasma triglycerides and whole-body insulin sensitivity into the inclusion criteria for MHO [81] will strengthen our classification of MHO pregnancies. Nonetheless, this study identifies morphometric and metabolic differences between placentae from MHO and normal weight pregnancies, providing valuable preliminary data to inform the development of new hypotheses.

## 5. Conclusions

Building on the theme of altered placental morphology and metabolomic profile in uncomplicated metabolically healthy obese (MHO) pregnancy, this study highlights how metabolomic profiling can aid in identifying pregnancy-related biomarkers, assessing placental and fetal well-being, predicting outcomes, and promoting personalized prenatal care [82]. Changes in several biochemical pathways, including cysteine and methionine and vitamin B6 metabolism, were among the most distinct signatures of MHO pregnancy. These metabolic changes underscore the practical value of assessing maternal metabolic health and its potential influence on the lifelong health of offspring. Nevertheless, further validation through larger cohort studies and broader biomarker sampling are warranted. The reported placental morphometric and metabolomic changes collectively establish a foundation for future research on screening and managing metabolically healthy obese (MHO) pregnancies. These findings emphasize the importance of controlling for the effects of obesity itself when studying metabolic markers in obesity-related pregnancy complications. Focusing on health markers, rather than relying solely on weight or BMI, is essential to fully capture the nuanced impact of maternal obesity on placental function and fetal development. A clear distinction between MHO and MUO pregnancies, as well as metabolically healthy and unhealthy states in non-obese populations, remains vital for advancing both research and clinical management strategies. Future studies should include and characterize MUO pregnancies to better understand the continuum of maternal metabolic health and its impact on placental function and offspring outcomes.

## Figures and Tables

**Table 1:** Placental morphometric and histopathological features in placentae from control (C, *n* = 15) participants and participants with metabolically healthy obesity (MHO, *n* = 15).

	C	MHO	*p*-Value	*d*/V
Placental weight ^1^ (g)	592.3 ± 111.0	762.0 ± 164.3	0.0026	1.2
Placental length (cm)	16.7 ± 2.1	18.1 ± 2.0	0.0685	0.7
Placental breadth (cm)	15.1 ± 1.5	16.9 ± 2.0	0.0091	1.0
Placental thickness (cm)	1.5 ± 0.4	2.0 ± 0.4	0.0049	1.2
Placental surface area (cm^2^)	199.6 ± 34.9	243.7 ± 52.7	0.0116	1.0
Distal villous hypoplasia (focal or diffuse) ^2^	0 (0)	0 (0)	NS	ND
Syncytial knots	0 (0)	0 (0)	NS	ND
Chorangiosis	0 (0)	2 (13)	NS	0.3
Delayed villous maturation (focal or diffuse)	1 (7)	5 (33)	NS	0.3
Avascular fibrotic villi	0 (0)	0 (0)	NS	ND
Increased focal perivillous fibrin deposition	1 (7)	1 (7)	NS	0.0
Massive perivillous fibrin deposition	0 (0)	0 (0)	NS	ND
Maternal floor infarct pattern	0 (0)	0 (0)	NS	ND
Intervillous thrombi	0 (0)	0 (0)	NS	ND
Chronic inflammation	0 (0)	0 (0)	NS	ND

^1^ placental morphometric data are presented as mean (±SD). BMI, body mass index. ^2^ distributions of different placental histopathological features are represented using frequency (*n*) and percentage in brackets (%). Effect size was calculated using Cohen’s *d* or Cramer’s V; NS = non-significant (*p* > 0.1); ND = not determined due to absence in both groups.

**Table 2:** Alterations in placental metabolites in the MHO vs. control groups.

**Compound ^1^**	**Fold Change**	***p* Adjusted**	***p*-Value**
3-Aminoisobutanoic acid (BAIBA)	0.6804	0.0011	<0.0001
2-Aminobutyric acid (2AB)	0.7071	0.0011	<0.0001
Gamma-glutamyl-glycine (γ-Glu-Gly)	0.8057	0.0198	0.0001
Ophthalmic acid (OPT)	0.6763	0.0448	0.0004
Prolyl-glutamine (Pro-Gln)	0.7571	0.0448	0.0003
N6-acetyl-LL-2,6-diaminoheptanedioic acid (N6-acetyl-LL-2,6-DAP)	0.8121	0.0448	0.0003
Valyl-asparagine (Val-Asn)	0.7438	0.0698	0.0007
Pyridoxal	0.6582	0.1130	0.0015
3-Hydroxykynurenamine O-sulfate (C05636)	0.7918	0.1967	0.0049
Pyridoxal phosphate (PLP)	0.8017	0.2440	0.0069

^1^ relative values of differentially expressed and identified metabolites in placentae from the MHO group, expressed as fold change of control placentae. Significantly different metabolites between the MHO and control group placentae (Fold change > 1.2 or <0.83 and FDR-corrected *p*-value < 0.25 (corresponding to a raw *p*-value threshold of 0.049) as criteria) are presented. *n* = 15 per group, with data from central, peripheral, and middle placental samples from each individual placenta combined.

**Table 3:** Pearson correlation (r) and *p*-values of linear regression for each metabolite with placental weight, thickness, breadth, and surface area.

**Metabolite**	**Placental** **Weight**		**Placental** **Thickness**		**Placental** **Breadth**		**Placental Surface Area**	
	**r**	***p***	**r**	***p***	**r**	***p***		
BAIBA	−0.2961	0.0046 *	−0.5406	<0.0001 *	−0.2792	0.0077 *	0.1339	0.2083
2-aminobutyric acid	−0.2927	0.0051 *	−0.5379	<0.0010 *	−0.2754	0.0086 *	−0.1359	0.2014
γ-Glu-Gly	−0.0939	0.3787	−0.3125	0.0075 *	−0.2448	0.0200 *	−0.1715	0.1060
Ophthalmic acid	−0.1531	0.1497	−0.2563	0.0298 *	−0.2283	0.0304 *	−0.1709	0.1073
Prolyl-glutamine	−0.1345	0.2061	−0.2930	0.0125 *	−0.1086	0.2061	−0.0696	0.5144
N6-acetyl-LL-2,6-DAP	−0.3018	0.0038 *	−0.4134	0.0003 *	−0.1559	0.1423	−0.0959	0.3688
Valyl-asparagine	−0.2324	0.0540 *	−0.3960	0.1569	−0.1282	0.0164 *	−0.1098	0.3030
Pyridoxal	−03432	0.0009 *	−0.2125	0.0731	−0.2640	0.0119 *	−0.1826	0.0849
C05636	−0.0625	0.5583	−0.0768	0.5211	−0.0168	0.8752	−0.0701	0.5115
Pyridoxal phosphate	−0.2298	0.0293 *	−0.2265	0.5580	−0.0720	0.4998	0.0406	0.7041

* statistical significance (*p* < 0.05); N = 15 per group (MHO vs. control groups). BAIBA = 3-aminoisobutanoic acid; γ-Glu-Gly = gamma-glutamyl-glycine; N6-acetyl-LL-2,6-DAP = N6-acetyl-LL-2,6-diaminoheptanedioic acid; C05636 = 3-hydroxykynurenamine O-sulfate.

## Data Availability

The raw data supporting the conclusions of this article will be made available by the authors upon request.

## Supplementary Materials

The following supporting information can be downloaded at: https://www.mdpi.com/article/10.3390/biomedicines13092149/s1, Table S1: Primer sequences used to measure mRNA expressions by RTq-PCR; Table S2: Reagents, Instruments, and Kits used in the study; Table S3: List of peak pairs detected from CIL LC-MS measurement of the samples (experimental data with imputed value); Figure S1: Workflow of sample processing and metabolomic analyses “Created in BioRender. Abdelwahab, N. (2025) https://BioRender.com/tud8ox0 (accessed on 24 July 2025)”.

## Abbreviations

AUC	Area Under the Curve
BPV	Birth Weight/Placental Volume Ratio
BPW	Birth Weight/Placental Weight Ratio
FDR	False Discovery Rate
GDM	Gestational Diabetes Mellitus
LC–MS	Liquid Chromatography–Mass Spectrometry
MHO	Metabolically Healthy Obesity
MUO	Metabolically Unhealthy Obesity
PLS-DA	Partial Least Squares Discriminant Analysis
ROC	Receiver Operating Characteristic
VIP	Variable Importance in Projection
CIL GC-MS	Chemical Isotope Labeling Gas Chromatography–Mass Spectrometry
QTOF	Quadrupole Time-of-Flight
