# Supplementary material for: Placental Morphology and Metabolomic Profile in Uncomplicated Metabolically Healthy Obese Pregnancy
Source: Biomedicines. 2025 Sep 3;13(9):2149. doi: 10.3390/biomedicines13092149 (PMC12467361; doi:10.3390/biomedicines13092149)
Supplement: Supplementary file 1 [file biomedicines-13-02149-s001.zip › Supplemental Figure S1.pdf]

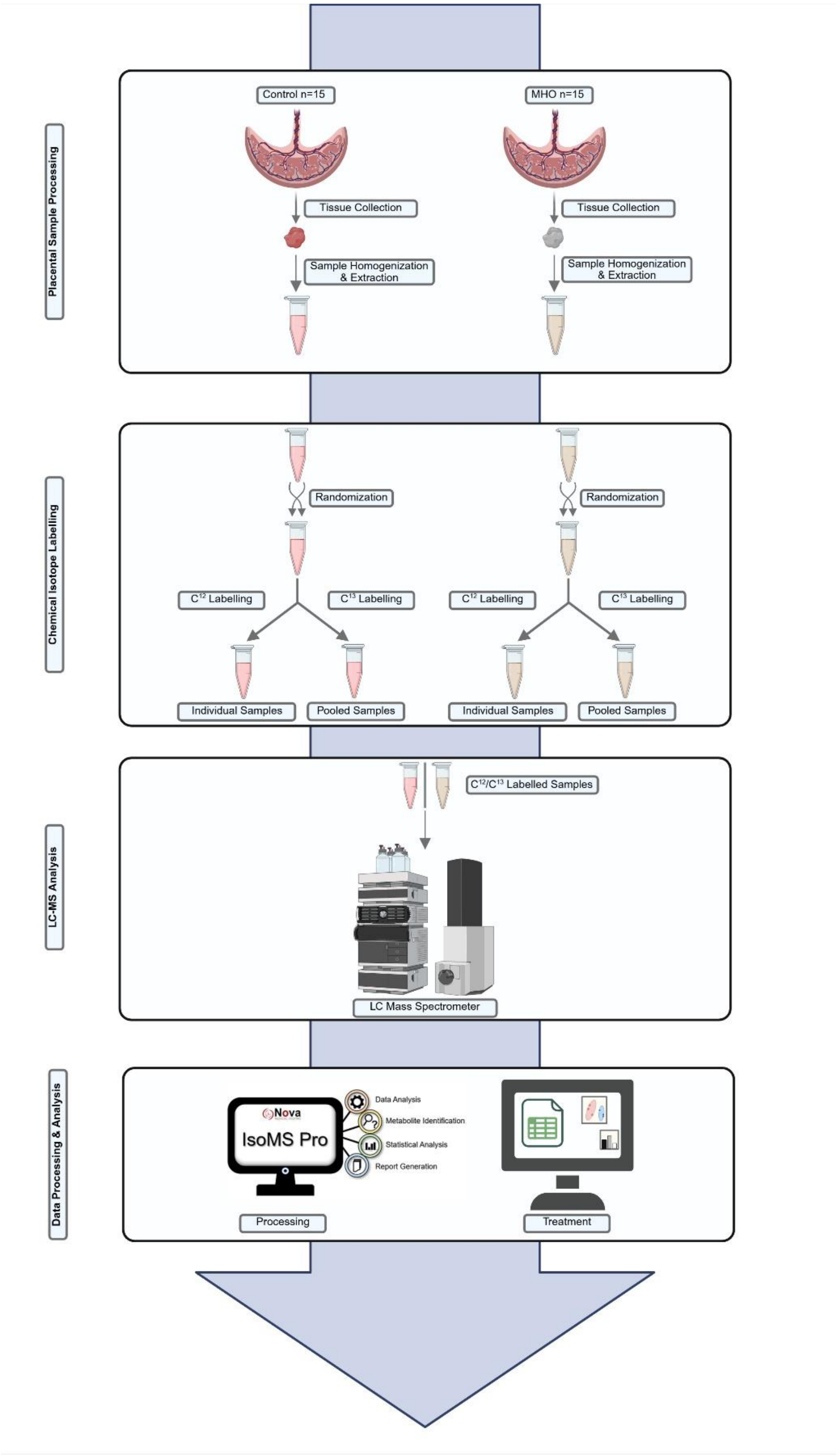

Supplemental Figure S1: Workflow of sample processing and metabolomic analyses "Created in BioRender. Abdelwahab, N. (2025) https://BioRender.com/tud8ox0 ".
