# Supplementary material for: Placental Morphology and Metabolomic Profile in Uncomplicated Metabolically Healthy Obese Pregnancy
Source: Biomedicines. 2025 Sep 3;13(9):2149. doi: 10.3390/biomedicines13092149 (PMC12467361; doi:10.3390/biomedicines13092149)
Supplement: Supplementary file 1 [file biomedicines-13-02149-s001.zip › Supplemental Table S1.pdf]

Table S1: Primer sequences used to measure mRNA expressions by RTq-PCR.

Gene	Accession number	Annealing temperature	Forward/reverse primer
SOD1	NM_000454.5	59 ºC	Forward: 5'-GGGGAAGCATTAAAGGACTG-3'<br>Reverse: 5'-CCACCGTGTTTTCTGGATAG-3'
SOD2	NM_001322820.2	62 ºC	Forward: 5'- GCGTTTACTCTTAGCAGAAGCTC-3'<br>Reverse: 5'- GGTGACGTTCAGGTTGTTCA-3'
CAT	NM_001752.4	64 ºC	Forward: 5'-GCCACAGGAAAGTACCCCTC-3'<br>Reverse: 5'- CGGTGAGTGTCAGGATAGGC-3'
GPX	NM_001329503.2	64 ºC	Forward: 5'-ACACCCAGATGAACGAGCTG-3'<br>Reverse: 5'-CAAACTGGTTGCACGGGAAG-3'
GSS	NM_000178.4	60 ºC	Forward: 5'-GGGGTATCCTCCTAAAGACC-3'<br>Reverse: 5'-CACTCAGTCCTATCCCAAGT-3'
GCLM	XM_047418031.1	60 ºC	Forward: 5'-GAAGAAGTGCCCGTCCA-3'<br>Reverse: 5'-GGTGAAGCAATGATCACAGA-3'
TNFα	NM_000594.4	60 ºC	Forward: 5'-AAACGGAGCTGAACAATAGG-3'<br>Reverse: 5'-ATTACAGACACAACTCCCCT-3'
IL6	NM_000600.5	60 ºC	Forward: 5'-CTTCGGTCCAGTTGCCTT-3'<br>Reverse: 5'-CCATCTTTGGAAGGTTCAGG-3'
IL10	NM_000572.3	62 ºC	Forward: 5'- TCTTGCAAAACCAAACCACAAGA-3'<br>Reverse: 5'- CCCAGGTAACCCTTAAAGTCC-3'
MCP1	NM_002982.4	58 ºC	Forward: 5'-CTCGCGAGCTATAGAAGAATC-3'<br>Reverse: 5'-TGTGGAGTGAGTGTTCAAGT-3'
TL3	NM_003265.3	60 ºC	Forward: 5'-ACTCCACCTCACTATCATGG-3'<br>Reverse: 5'-TCCCAGACCCAATCCTTATC-3'
Β-actin	NM_001101.5	63 ºC	Forward: 5'-GTTGCTATCCAGGCTGTGCT-3'<br>Reverse: 5'-AGGTAGTCAGTGAGGTCCCG-3'
PMSB6	NM_002798.3	76 ºC	Forward: 5'-CGGGAAGACCTGATGGCGGGA-3'<br>Reverse: 5'-TCCCGGAGCCTCCAATGGAAA-3'
