# Supplementary material for: Placental Morphology and Metabolomic Profile in Uncomplicated Metabolically Healthy Obese Pregnancy
Source: Biomedicines. 2025 Sep 3;13(9):2149. doi: 10.3390/biomedicines13092149 (PMC12467361; doi:10.3390/biomedicines13092149)
Supplement: Supplementary file 1 [file biomedicines-13-02149-s001.zip › Supplemental Table S2.pdf]

Supplemental Table S2: Reagents, Instruments, and Kits Used in the Study

Item/Reagent/Instrument	Manufacturer	Catalog or CAS Number/ Software version
Aperio ScanScope CS	Leica, Microsystems CMS GmbH	DM500 or DM3000?
Methanol	Canadian Life Science	HW-34966-4X4L-AZ001
Dichloromethane	Canadian Life Science	HW-650463-4X4L
Metabolome Quantification kit	Nova Medical Testing Inc	NMT-6001-KT
Agilent 1290 Infinity LC	Agilent	1290 Infinity LC
Bruker Impact II QTOF Mass Spectrometer	Bruker	Impact II
ZORBAX RR Eclipse Plus C18 Reversed-Phase HPLC Column	Agilent	959963-902
Formic acid	Fisher scientific	A11750
Acetonitrile	Canadian Life Science	HW-34967-4X4L-AZ001
DataAnalysis	Bruker Daltonik GmbH	Version 4.4
IsoMS Pro	Institute for Systems Biology (ISB)	Version 1.2.12
NovaMT Metabolite Databases	Nova Medical Testing Inc	Version 2.0
Labeled Metabolite Library (CIL Library)	Nova Medical Testing Inc	Version 1.0
Identity Library (LI Library)	Nova Medical Testing Inc	Version 1.0
MyCompoundID (MCID) Library	www.mycompoundid.org (Wishart Research Group, University of Alberta)	–
Trizol	Invitrogen	15596026
E.Z.N.A Total RNA kit I	OMEGA bio-tek	R6834-01
Nanodrop spectrophotometer	Thermo Fisher Scientific	ND-2000
M-MLV Reverse Transcriptase Kit	Thermo Fisher Scientific	18057018
SensiFAST SYBR No-ROX Kit	FroggaBio	BIO-98050
CFX Maestro software	Bio-Rad	Version 2.3
HEPES	Sigma-Aldrich	7365-45-9
sucrose	BioShop	57-50-1

Item/Reagent/Instrument	Manufacturer	Catalog or CAS Number/ Software version
EGTA	Sigma-Aldrich	13368-13-3
Mannitol	Thermo Fisher Scientific	Catalog No.
Catalase assay kit	Cayman Chemical	707002
Bio-Gen PRO200 Homogenizer	PRO Scientific Inc.	PRO200
Superoxide dismutase assay kit	Cayman Chemical	706002
Glutathione Assay Kit	Cayman Chemical	703002
8-isoprostane enzyme-linked immunosorbent assay kit	Cayman Chemical	516351
EDTA	Sigma-Aldrich	60-00-4
Butylated Hydroxytoluene (BHT)	Sigma-Aldrich	128-37-0
Potassium hydroxide (KOH)	Sigma-Aldrich	1310-58-3
Hydrochloric Acid (HCl)	Sigma-Aldrich	7647-01-0
SEP cartridge C18 columns	Thermo Fisher Scientific	03-251-258
TBARS Assay	Cayman Chemical	10009055
NaCl	Sigma-Aldrich	7647-14-5
Triton X-100	Sigma-Aldrich	9036-19-5
Sodium deoxycholate	Sigma-Aldrich	302-95-4
Sodium dodecyl sulfate	Sigma-Aldrich	151-21-3
Na_3V0_4	Sigma-Aldrich	S6508
NaF	Thermo Fisher Scientific	7681-49-4
PMSF	Sigma-Aldrich	329-98-6
Leupeptin	Sigma-Aldrich	103476-89-7
Aprotinin	Sigma-Aldrich	9087-70-1
GraphPad Prism	GraphPad Software, LLC	9.0.0
MetaboAnalyst	www.metaboanalyst.ca (Xia Lab, McGill University)	5.0
IBM SPSS Statistics	IBM Corporation	28.0
